# *Methyl-CpG binding protein 2* expression is associated with symptom severity in patients with PTSD in a sex-dependent manner

**DOI:** 10.1038/s41398-023-02529-9

**Published:** 2023-07-07

**Authors:** Livia Cosentino, Stephanie H. Witt, Helene Dukal, Francesca Zidda, Sebastian Siehl, Herta Flor, Bianca De Filippis

**Affiliations:** 1 Center for Behavioral Sciences and Mental Health, Istituto Superiore di Sanità, Roma, Italy; 2 Institute of Cognitive and Clinical Neuroscience, Central Institute of Mental Health, Medical Faculty Mannheim, Heidelberg University, Mannheim, Germany; 3 Department of Genetic Epidemiology in Psychiatry, Central Institute of Mental Health, Medical Faculty Mannheim, Heidelberg University, Mannheim, Germany; 4 Institute of Medical Psychology and Medical Sociology, University Medical Center Schleswig-Holstein, Kiel University, Kiel, Germany

**Keywords:** Clinical genetics, Psychiatric disorders, Diagnostic markers

## Abstract

Traumatic events may lead to post-traumatic stress disorder (PTSD), with higher prevalence in women. Adverse childhood experiences (ACE) increase PTSD risk in adulthood. Epigenetic mechanisms play important roles in PTSD pathogenesis and a mutation in the *methyl-CpG binding protein 2* (*MECP2*) in mice provide susceptibility to PTSD-like alterations, with sex-dependent biological signatures. The present study examined whether the increased risk of PTSD associated with ACE exposure is accompanied by reduced *MECP2* blood levels in humans, with an influence of sex. *MECP2* mRNA levels were analyzed in the blood of 132 subjects (58 women). Participants were interviewed to assess PTSD symptomatology, and asked to retrospectively report ACE. Among trauma-exposed women, *MECP2* downregulation was associated with the intensification of PTSD symptoms linked to ACE exposure. *MECP2* expression emerges as a potential contributor to post-trauma pathophysiology fostering novel studies on the molecular mechanisms underlying its potential sex-dependent role in PTSD onset and progression.

## Introduction

Trauma exposure is a common experience worldwide, with 70% of people exposed to at least one traumatic event throughout their lives [1]. This may lead to the onset of post-traumatic stress disorder (PTSD), a chronic mental disorder characterized by severely debilitating and long-lasting symptoms, that is more prevalent in women than men [2]. PTSD symptoms can be grouped in four main categories [3]: (i) re-experiencing, defined as the appearance of intrusive thoughts, nightmares and flashbacks; (ii) avoidance of internal or external trauma reminders; (iii) hyperarousal, manifested as attentional threat bias, sleep problems and enhanced startle reactivity; (iv) negative alterations in cognition and mood, including patients’ inability to recall important aspects of the traumatic event and persistent negative emotional state. Only an average of 5.6% of traumatized individuals in the world develop a chronic PTSD symptomatology [4]. Nevertheless, the high socio-economic costs and the burden that PTSD symptomatology represents for the affected individuals urge the identification of the risk factors involved in disease development in traumatized people [5].

In recent years the neurobiological bases of PTSD have been deeply explored [6], and a growing body of evidence has underscored the contribution of epigenetic mechanisms to PTSD pathogenesis and symptom presentation in the aftermath of trauma exposure [7]. Among the multiple epigenetic signatures, altered DNA methylation has been especially linked to traumatic stress consequences [8]. Both candidate gene and epigenome-wide association studies have in fact identified PTSD-related alterations in methylated DNA (mDNA) at multiple genetic *loci* involved in stress, inflammation and neurotransmission pathways [9–11]. On these grounds previous works also found variations in the enzymes responsible for DNA methylation in association with risk for PTSD [12]. In spite of this evidence, the potential role in PTSD pathophysiology of mDNA reader binding proteins, such as the X-linked methyl-CpG binding protein 2 (MECP2), has not yet been addressed. MECP2 in particular serves as a scaffold protein for the recruitment of chromatin remodeling complexes [13–15] and DNA methyltransferases [16, 17] on methylated DNA *loci*, thus representing an excellent candidate for mediating post-trauma epigenetic rearrangements. Moreover, the activity and expression of MECP2 in the rodent brain, where MECP2 is known to regulate learning and memory processes [18], is very responsive to environmental challenges such as exposure to early life stress as well as to stressors in adulthood [19–21]. In this line, previous studies searching for genetic markers of PTSD risk described an altered expression of multiple targets of MECP2 in subjects who developed the disorder in the aftermath of a traumatic experience [22–25]. Consistently, MECP2 is known to control the transcription of stress response-regulating genes [26, 27], and to tune immune function and cytokine production [28], whose alterations have been described in patients with PTSD [22, 29–33]. These characteristics make MECP2 a promising mediator of the lasting epigenetic adjustments taking place following stress or trauma exposure that could direct towards vulnerability or resilience to PTSD [34]. Based on this, we recently addressed the potential involvement of altered MECP2 functionality in the onset of PTSD-like pathophysiology in transgenic mice carrying a hypofunctional form of *MECP2*. We demonstrated that *MECP2*-mutated mice display an increased propensity to develop enduring neurobehavioral alterations, comparable to those observed in patients with PTSD, when exposed to intense, acute stressors [35].

Notably, male and female carriers of the *MECP2* hypomorphic mutation, while being both behaviorally sensitive to stressors, exhibited an opposite modulation of stress markers at the molecular level [36]. This finding opens up the intriguing possibility that the MECP2 protein may be involved in the regulation of sex-dependent differences in vulnerability to PTSD. In this line, it is noteworthy that MECP2 has been proposed to participate in the sexual differentiation of the developing rodent brain [37, 38], suggesting that it might play a role in setting the basis for the existing sex bias in vulnerability to PTSD [39, 40].

Interestingly, a sex-specific modulation of *MECP2* expression has previously been described in rodents following stress exposure early in life [41, 42]. Furthermore, in a non-clinical population sample we recently demonstrated that reduced levels of *MECP2* are linked to an increased risk of psychopathology following childhood adversities selectively in women [43]. Given that early-life adversities, vulnerability to psychopathology and female sex are factors known to intensify the impact of exposure to traumatic experiences in adulthood, eventually increasing the risk of PTSD onset [44, 45], these findings confirm the need of further exploring the role of *MECP2* in the pathogenesis of PTSD.

Based on this body of evidence, we hypothesized that *MECP2* levels might be altered within a traumatized population, with its downregulation possibly representing a risk factor for developing PTSD. As exposure to stressful experiences at critical developmental periods, such as childhood, dramatically increases vulnerability to the pathological outcomes of subsequent trauma exposure, we explored the possibility that adverse childhood experiences (ACE) strengthen the association between reduced *MECP2* and an increased PTSD risk. We also reasoned that this association might be more marked in women, in line with the existing sex bias in vulnerability to PTSD. To test our assumptions we evaluated *MECP2* mRNA levels in the blood of 132 male and female participants who were trauma-exposed with or without ensuing symptoms of PTSD in adulthood (PTSD and trauma controls, TC) or non-exposed throughout their lifetime (non-traumatized controls, NTC), and assessed whether *MECP2* expression varied as a function of sex, ACE and trauma exposure. Focusing on traumatized individuals, we tested the possibility that the increased severity of PTSD symptoms associated with exposure to ACE was accompanied by reduced *MECP2* blood levels, with an influence of sex.

## Materials and methods

### Study participants

Study participants were civilians recruited between 2010 and 2018 to take part in multiple independent studies on the psychobiological alterations characterizing people suffering from PTSD (see Supplementary Methods for further information). A total of 132 subjects (58 women, mean age 41.72 ± 13.91 years) were included in the present study; among them, 85 subjects reported traumatic experiences, of whom 37 received a diagnosis of PTSD (see Table 1 for detailed information on the study sample). PTSD diagnosis and possible comorbidities were evaluated by the Structured Clinical Interviews for DSM-IV [46] I and II (SCID) [47, 48] (see Supplementary Methods). Participants were excluded in case of clinically significant traumatic experiences before 18 years of age, comorbid psychotic symptoms, borderline personality disorder, alcohol/drug dependence or abuse, and cardiovascular or neurological disorders. The study conformed to the Code of Ethics of the World Medical Association (Declaration of Helsinki, 6^th^ revision, 2008) and was approved by the Ethics Committee of the Medical Faculty Mannheim, Heidelberg University. All participants gave written informed consent.

**Table 1:** Demographic and clinical information.

Variable		Group							Group statistic
			NTC (*N* = 47)		TC (*N* = 48)		PTSD (*N* = 37)		
			Men (*N* = 29)	Women (*N* = 18)	Men (*N* = 25)	Women (*N* = 23)	Men (*N* = 20)	Women (*N* = 17)	
**Age (in years)**		Mean (SD)	40.07 (14.16)	45.53 (15.96)	42.04 (14.38)	43.13 (16.35)	39.70 (9.88)	40.71 (11.99)	Diagnosis: F_2,125_ = 0.41, *p* = 0.66 Gender: F_1,125_ = 1.01, *p* = 0.32
**Education**	≤ 12 years	N	0	3	2	1	4	4	χ^2^_15_ = 18.38, *p* = 0.24
	> 12 years	N	28	14	20	20	13	13	
	Other	N	1	1	1	0	1	0	
	Missing	N	0	0	2	2	2	0	
**Ancestry**	European	N	26	17	22	21	14	14	χ^2^_10_ = 11.06, p = 0.35
	Other^a^	N	3	1	2	0	3	2	
	Missing	N	0	0	1	2	3	1	
**CES-D**		Mean (SD)	7.21 (4.22)	7.50 (5.83)	10.04 (6.99)	10.70 (8.11)	31.10 (9.16)	29.06 (10.03)	Diagnosis: F_2,126_ = 109.45, *p* < 0.001 (PTSD > NTC & TC) Gender: F_1,126_ = 0.08, *p* = 0.78
**STAI-T**		Mean (SD)	32.24 (9.38)	31.61 (6.46)	35.83 (8.25)	35.91 (11.69)	58.58 (9.79)	52.71 (11.53)	Diagnosis: F_2,123_ = 66.88, *p* < 0.001 (PTSD > NTC & TC) Gender: F_1,123_ = 1.54, *p* = 0.22
**Medication**	None	N	13	8	16	6	2	6	χ^2^_15_ = 41.10, *p* < 0.001
	Psychopharmacological^b^	N	2	2	2	2	7	7	
	Non-psychopharmacological^c^	N	11	6	6	12	4	4	
	Missing	N	3	2	1	3	7	0	
**CTQ**		Mean (SD)	31.32 (8.52)	32.61 (12.82)	33.12 (10.04)	35.87 (17.06)	36.65 (13.24)	48.18 (22.57)	Diagnosis: F_2,126_ = 5.88, *p* = 0.004 (PTSD > NTC & TC) Gender: F_1,126_ = 4.31, *p* = 0.040 (F > M)
**TICS**		Mean (SD)	2.07 (0.56)	2.04 (0.47)	2.34 (0.78)	2.19 (0.65)	3.04 (0.58)	2.81 (0.59)	Diagnosis: F_2,120_ = 20.38, *p* < 0.001 (PTSD > NTC & TC) Gender: F_1,120_ = 1.43, *p* = 0.23
**Time since trauma (in years)**		Mean (SD)	-	-	9.23 (8.51)	11.80 (12.41)	10.38 (9.09)	8.91 (11.06)	Diagnosis: F_1,72_ = 0.13, *p* = 0.72 Gender: F_1,72_ = 0.05, *p* = 0.82
**Index trauma**	Caused voluntarily^d^	N	-	-	11	6	12	12	χ^2^_6_ = 11.07, *p* = 0.09
	Caused involuntarily^e^	N	-	-	14	16	7	5	
	Missing	N	-	-	0	1	1	0	
**CAPS**		Mean (SD)	-	-	7.74 (8.84)	9.37 (11.40)	67.18 (23.42)	65.91 (20.53)	Diagnosis: F_1,74_ = 235.44, *p* < 0.001 (PTSD > TC) Gender: F_1,74_ = 0.002, *p* = 0.96
**PDS**		Mean (SD)	-	-	8.04 (7.17)	9.00 (10.87)	36.35 (7.77)	32.13 (8.93)	Diagnosis: F_1,75_ = 173.44, *p* < 0.001 (PTSD > TC) Gender: F_1,75_ = 0.66, *p* = 0.42

*Abbreviations:* *NTC* Non-traumatized controls, *TC* Traumatized controls, *PTSD* Patients diagnosed with post-traumatic stress disorder, *SD* Standard deviation, *N* Sample size, *χ*^*2*^ Chi square statistic, *F* F statistic, *p* p value of the chi square or F statistic, *CES-D* Center for Epidemiological Studies Depression Scale, German version, *STAI-T* Trait scale of the State-Trait Anxiety Inventory, *CTQ* Childhood trauma questionnaire, *TICS* Trier Inventory for Chronic Stress, *CAPS* Clinician-Administered PTSD Scale, *PDS* Post-traumatic Diagnostic Scale, *Symbols:* ^a^ Mixed, Turkish, Columbian, Pakistani, Palestinian; ^b^ Aripiprazole, Pregabalin, Methylphenidate, Mirtazapine, Quetiapine, Sertraline, Trimipramine, Venlafaxine; ^c^ Etoricoxib, Bisoprolol, Beta-Blocker, Ibuprofen, Metamizole, Levothyroxine; ^d^ Imprisonment, torture, physical violence, sexual abuse/rape, wartime experience, witness of sudden death/serious injury of someone, other experience; ^e^ fire or explosion, accident, sudden death of someone, other experiences.

### Psychometric measures

#### Posttraumatic Diagnostic Scale

Traumatic experiences were assessed by the means of the German version of the Posttraumatic Diagnostic Scale (PDS) [49, 50], a self-report instrument aimed at assessing the severity of post-traumatic stress symptoms. The first part of the questionnaire consists of a short checklist of potentially traumatizing events. Among the experienced events, respondents are required to indicate the one that has troubled them the most in the past month (index trauma). Participants experiencing the index trauma before 18 years of age were excluded, since clinically significant childhood trauma experiences are expected to have differential impacts on PTSD pathophysiology [22]. The subjects reporting an index trauma were then required to rate, on a 4-point scale (0 - never to 3 - daily), 17 items representing the frequency of the occurrence of cardinal PTSD symptoms in the last 30 days. Finally, respondents rated the degree of impairment caused by symptoms across different areas of life functioning. The symptom severity score was obtained by adding up the responses to selected items and ranges from 0 to 51.

#### Clinician-Administered PTSD Scale

PTSD symptomatology was assessed by the means of the Clinician-Administered PTSD Scale interview (CAPS) [51, 52], a 30-item structured interview that corresponds to the DSM-IV criteria for PTSD [46]. Frequency and severity of each item are rated on a 5-point Likert scale ranging from 0, never/not affected to 4, every day/extremely affected. Three subscales measuring re-experiencing, avoidance and arousal symptom clusters were then calculated as the mean frequency and severity values of the relative items (5 for re-experiencing and arousal and 7 for avoidance symptoms). The total CAPS score was calculated as the overall sum of the ratings, ranging from 0 to 68.

#### Adverse childhood experiences

Participants completed the Childhood Trauma Questionnaire [53] in order to retrospectively evaluate the severity of ACE (<18 years of age), including emotional, physical abuse/neglect and sexual abuse. This is a psychometrically validated self-report inventory composed of 28 items each rated on a 5-point Likert scale (1, never true – 5, very often true). The total score ranges from 25 to 125. We exploited the classification of the total score into *severity quartiles* (*none/minimal*, *low to moderate*, *moderate to severe*, *severe to extreme*) contained in the manual (Bernstein & Fink, 1998; MacDonald et al., 2016) to include ACE as a discrete independent factor in tests of analysis of variance (ANOVA). By merging *quartiles 2-4* we obtained a dichotomous variable: 1, *none/minimal* (total score ≤ 36) - for individuals who did not report ACE, and 2, *low to extreme* (total score > 36) - for individuals who recalled ACE of different intensities.

#### Trier Inventory for Chronic Stress

The load of current chronic stress was assessed by the means of the Trier Inventory for Chronic Stress (TICS) [54], a self-assessment instrument composed of 57 items evaluating 9 chronic stressors: work and social overload, pressure to perform, work discontent, excessive demand at work, lack of social recognition, social tensions or isolation and chronic worrying. Each item is rated on a 5-point Likert scale indicating how often the subject had experienced a certain situation within the last 3 months (0, never – 4, very often).

#### Center for Epidemiological Studies Depression Scale and State-Trait Anxiety Inventory

See Supplementary Methods.

### *MECP2* expression

Whole blood was collected in PAXgene Blood RNA Tubes (PreAnalytiX, Hombrechtikon Switzerland) and stored until analysis at −80 °C [43, 55]. A PAXgene Blood miRNA Kit (Qiagen, Hilden, Germany) was used to extract total RNA, following the manufacturer´s instructions. RNA concentration and sample purity were assessed with a NanoDrop 1000 Spectralphotometer (Thermo Scientific, Waltham, MA, USA), and RNA integrity was determined with the Agilent 2100 Bioanalyzer System (Agilent Technologies, Santa Clara, CA, USA). The cDNA was synthesized by a reversed transcription reaction using the High Capacity cDNA Reverse Transcription Kit (Applied Biosystems, Waltham, MA, USA). Quantitative PCR was performed on the QuantStudio 7 Flex Real-Time PCR System (Applied Biosystems by Life Technologies, Carlsbad, CA, USA) by using TaqMan Fast Advanced Mastermix (Applied Biosystems), and the *MECP2* TaqMan Gene Expression Assay Hs00172845_m1 (Applied Biosystems). The Actin Beta *ACTB* TaqMan Gene Expression Assay Hs01060665_g1 (Applied Biosystems) was used as an internal standard. Results were calculated with the QuantStudio Real-Time PCR Software v1.3 (Applied Biosystems by Thermo Fisher Scientific). Analyses were carried out in triplicates. All data were normalized to the endogenous reference gene *ACTB*. For statistical analyses, the relative expression with respect to participants not reporting traumas or ACE (controls) was calculated by the Delta-Delta threshold cycles (∆∆Ct) method, and converted to the relative expression ratio (2^-∆∆Ct^), separately for men and women [56].

### Statistical analyses

All statistical analyses were conducted using SPSS 20.0 and AMOS 20.0 (IBM Statistics, Armonk, NY, USA).

A logarithmic transformation was performed to reduce skewedness and kurtosis of non-normally distributed variables (see Supplementary Methods and Table S1). Outliers, defined as observations lying three standard deviations outside from the mean, were excluded (2 observations, in total).

A three-way ANOVA was performed to evaluate the relative role of sex (men & women), ACE (none/minimal & low to extreme) and trauma exposure (non-traumatized & traumatized in adulthood) in the modulation of peripheral *MECP2* expression. Normality and homoscedasticity of residuals were assessed by the means of Shapiro Wilk, Levene and Breush Pagan tests. *Post hoc* comparisons were performed by Tukey’s test.

Structural Equation Modeling (SEM) with maximum likelihood estimation was used to test the hypothesis that reduced *MECP2* expression is associated with the increased risk of developing PTSD following an index trauma, as the result of previous exposure to ACE. Exclusion criteria for the model were: failure to converge after 240 iterations, the presence of squared multiple correlation values greater than 1 (R^2^ > 1) and poor fit, estimated via the following goodness-of-fit (GOF) measures: the χ^2^statistic (with a good fit indicated by χ^2^/degrees of freedom (df) < 3), the root mean square error of approximation (RMSEA, with a good fit indicated by an index < 0.08) and the comparative fit index (CFI, with a good fit indicated by an index > 0.95) [57]. To establish mediation, indirect paths were tested for significance using a Bias-Corrected (BC) Bootstrapping method (95% confidence intervals; 2000 resamples) [58]. At least 10 observations per measured variable were included [59]. We checked whether the final model predicted equally well PTSD symptoms while using different scales established in the literature (CAPS and PDS). To examine whether the final model was specific for ACE, it was retested with a measure of current perceived chronic stress replacing the ACE score.

To dissect the moderating role of sex, the model was separately re-specified on male and female subsamples. For each of the analyses the alpha level was set to 0.05 [60, 61].

## Results

### *MECP2* expression reflects exposure to adversities in childhood and traumas in adulthood as a function of sex

**Fig. 1:**
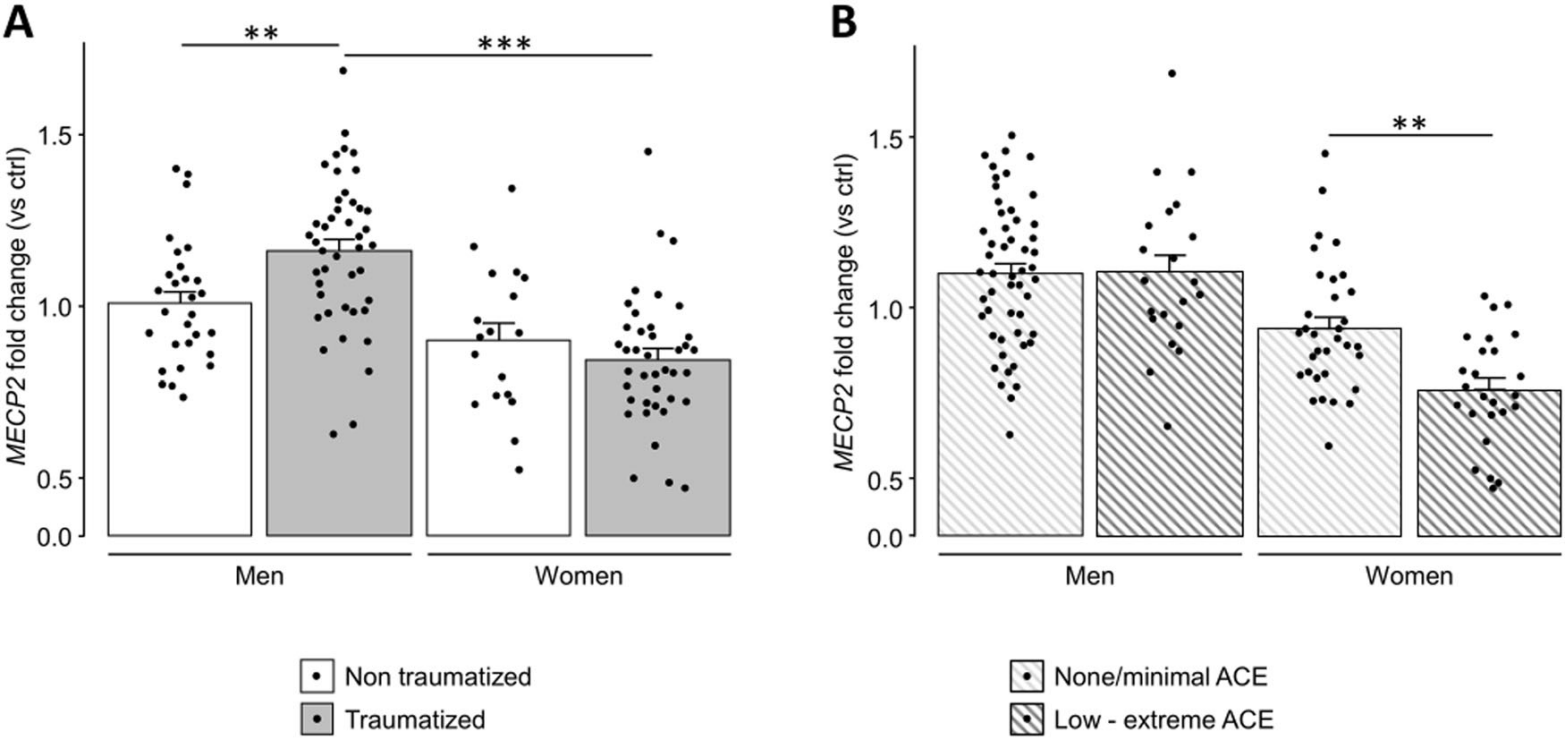
*MECP2* is overexpressed in traumatized men and underexpressed in women exposed to childhood adverse experiences. **A** Blood mRNA levels of *methyl-CpG binding protein 2* (*MECP2*) are increased in traumatized men, compared to non-traumatized men and women. **B** *MECP2* is downregulated in the blood of women exposed to adverse childhood experiences (ACE) compared to non-stressed women and men. *MECP2* levels were normalized to total *actin beta* (*ACTB*) contents and expressed as a proportion of those of non-traumatized participants, not exposed to ACE (ctrl), separately for men and women. Statistical significance was calculated by the means of three-way ANOVA, and Tukey’s *post hoc* tests. *Symbols:* **p < 0.01; ***p < 0.001. Data are mean ± standard error of the mean.

To evaluate whether exposure to traumatic experiences in adulthood or a history of ACE are associated with an altered peripheral expression of *MECP2*, and to test the moderating effect of sex, we exploited a three-way ANOVA model. The levels of blood *MECP2* mRNA were significantly higher in men compared to women (sex: F_1,124_ = 37.89, *p* < 0.001, η_p_^2^ = 0.23). This difference was driven by a sex-dependent effect of trauma exposure, which was associated with an increase of *MECP2* expression selectively in men (*p* = 0.009 for non-traumatized vs traumatized men after *post hoc* comparisons on an almost significant sex*trauma interaction: F_1,124_ = 3.79, *p* = 0.054, η_p_^2^ = 0.03; Fig. 1A) without affecting women. Conversely, peripheral *MECP2* expression was significantly decreased in participants reporting ACE (ACE: F_1,124_ = 7.46, *p* = 0.007, η_p_^2^ = 0.06), especially among women (p = 0.006 for women with none/minimal vs low to extreme ACE after *post hoc* comparisons on sex*ACE interaction: F_1,124_ = 7.21, *p* = 0.008, η_p_^2^ = 0.06; Fig. 1B). The three-way interaction between sex, ACE and index trauma was not significant (sex*ACE*trauma: F_1,124_ = 2.32, *p* = 0.130, η_p_^2^ = 0.02).

### Reduced *MECP2* expression accompanies the increase in the severity of PTSD symptoms associated with ACE exposure in traumatized participants

**Fig. 2:**
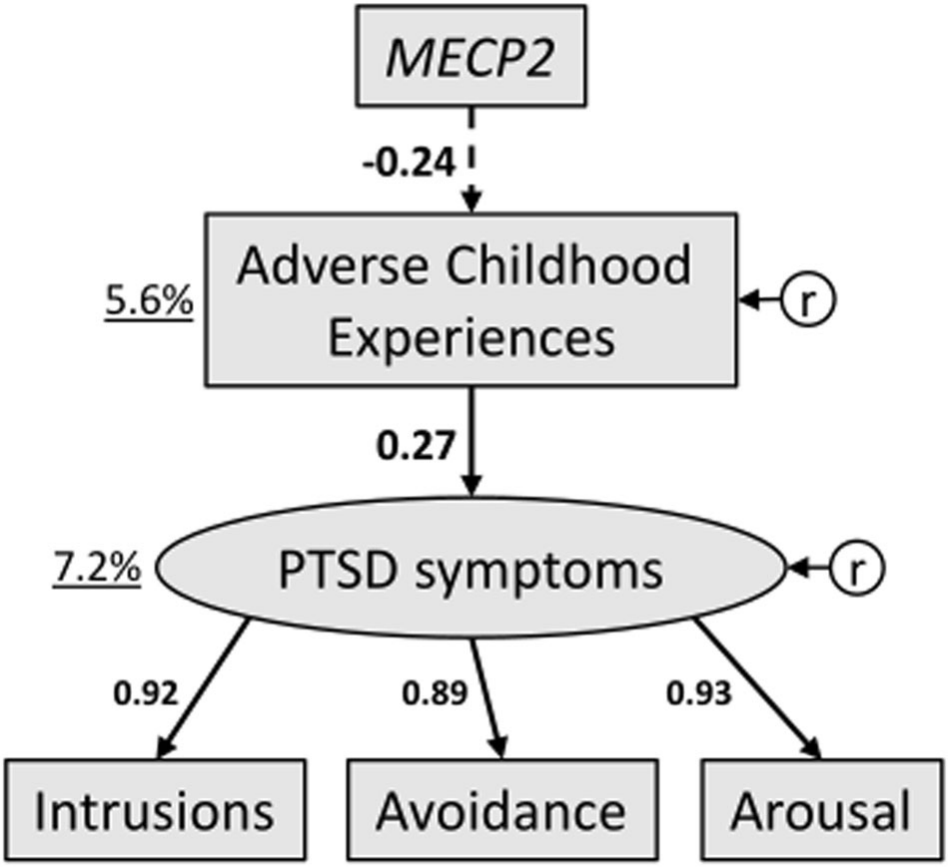
The increased severity of PTSD symptoms, which in traumatized participants is associated with exposure to adverse childhood experiences, is accompanied by reduced *MECP2* levels. Among traumatized participants, reduced *methyl-CpG binding protein 2* (*MECP2*) expression is directly associated with higher adverse childhood experiences (ACE) scores (R^2^ = 5.6%; β = −0.24, *p* = 0.037), which in turn predict increased post-traumatic stress disorder (PTSD) symptom severity (R^2^ = 7.4%; β = 0.27, *p* = 0.015). PTSD symptom severity is measured by means of the clinician-administered PTSD scale (CAPS). *Symbols:* Plain arrows - directed arcs, positive path coefficients (*p* < 0.05); dashed arrows - directed arcs, negative path coefficients (*p* < 0.05); black numbers - standardized coefficients; black underlined numbers – proportion of total variation explained by the model (R^2^); r – residual variances (errors).

Given that ACE are known to increase the risk of PTSD onset in the aftermath of traumatic events, using structural equation modeling we tested the hypothesis that the increase in the severity of PTSD symptoms (total CAPS score) associated with ACE exposure in individuals reporting an index trauma is accompanied by reduced *MECP2* levels (Fig. 2). Overall PTSD symptomatology was represented as a single latent factor in the present model, based on the a priori assumption that the three PTSD symptom subscales may be all associated within the same latent construct [51, 62].

The model fit was satisfactory (Tables 1, 2), suggesting that the hypothesized path (Fig. 2) describes the data well, thus allowing further interpretations of the results to be obtained. *MECP2* expression was inversely proportional to ACE severity (R^2^ = 5.6%), implying that decreased *MECP2* levels were associated with a stressful childhood (β = −0.24, *p* = 0.037). As expected, higher ACE scores were also linked to increased PTSD symptom severity (R^2^ = 7.2%) among traumatized individuals (β = 0.27, *p* = 0.022). Overall, *MECP2* expression turned out to be indirectly associated with PTSD symptoms, with ACE mediating the association between lower levels of *MECP2* and higher total CAPS scores (β = −0.06, *p* = 0.037). This suggests that *MECP2* downregulation accompanies the increased PTSD vulnerability emerging from a history of ACE (see Table 3 and Supplementary Table S2 for further details on direct and indirect effects in the hypothesized model).

**Table 2:** Goodness of fit indices.

Model	*Ν*	χ^2^	df	χ^2^/df	RMSEA	SRMR	TLI	CFI
(1) Hypothesized model	75	1.78	5	0.36	0	0.02	1.03	1
(2) Confirmatory models								
(i) PTSD symptom scale substitution model	78	2.31	5	0.46	0	0.03	1.03	1
(ii) Chronic stress model	74	7.44	5	1.49	0.08	0.05	0.98	0.99
(iii) Men	40	1.41	5	0.28	0	0.06	1.06	1
(iv) Women	35	5.59	5	1.12	0.06	0.08	0.99	0.99

*Abbreviations: N* Sample size, *χ*^*2*^ chi square statistic, *df* Degrees of freedom, *RMSEA* Root mean square error of approximation, *SRMR* Standardized root mean square residual, *TLI* Tucker-Lewis index, *CFI* Comparative fit index.

**Table 3:** Indirect effects of *MECP2* expression on PTSD symptomatology in the hypothesized and confirmatory models.

*MECP2* → PTSD symptoms (latent construct)	b (SE)	CI	β	p-value
Hypothesized model	−0.20 (0.14)	−0.58 to −0.01	−0.06	0.037
PTSD symptom scale substitution model	−0.45 (0.21)	−0.91 to −0.11	−0.12	0.009
Chronic stress model	0.07 (0.19)	−0.33 to 0.45	0.02	0.685
Men subsample	−0.003 (0.138)	−0.36 to 0.26	−0.001	0.950
Women subsample	−0.63 (0.33)	−1.47 to −0.10	−0.14	0.033

*Abbreviations:* *MECP2* Methyl-CpG binding protein 2, *PTSD* Post-traumatic stress disorders, *b* Unstandardized coefficient, *SE* Standard error, *CI* 95% confidence intervals for b, *β* Standardized coefficient, *Symbols:* underlined – significant results.

Importantly, the present results were further confirmed after retesting the hypothesized path on the prediction of PTSD symptom severity measured through a distinct psychometric scale (total PDS score, R^2^ = 19.2%) (PTSD *symptom scale substitution model*, see Tables 2, 2i, Table 3 and Supplementary Table S3 for detailed statistical results, and Supplementary Fig. S1).

### The increase in PTSD symptomatology associated with current stress load is not paralleled by changes in *MECP2* expression

To examine the specificity of the observed effects for stress experienced during childhood, we assessed whether the hypothesized path was still valid when replacing the ACE score with a measure of current chronic stress load (Supplementary Fig. S1).

The model had acceptable fit indices (Table 2, 2ii), and explained a relatively high proportion of total PTSD symptom variation (R^2^ = 26%), which was due to the highly significant association between chronic stress load and PTSD symptoms (β = 0.51, *p* < 0.001) (see Supplementary Table S4 for further information on direct and indirect effects). Indeed, *MECP2* was not significantly associated with chronic stress and failed to have significant indirect effects on PTSD symptomatology in the present model (see Table 3), suggesting that the association between *MECP2* expression and PTSD symptomatology is indirectly mediated specifically by stressors experienced during childhood.

### The link between *MECP2* downregulation and the increase in the severity of PTSD symptoms associated with ACE exposure is particularly relevant in women

In order to dissect the effects of sex, we explored the validity of the selected path on two different subsamples, composed of men or women only (Supplementary Fig. S2). The GOF indices for both subsamples were acceptable (see Table 2, 2iii and 2iv). In terms of R^2^ the model explained up to 27.8% of PTSD symptom variance in the female subsample, but failed to significantly explain PTSD symptom variance in the male subsample.

Importantly, in both samples, *MECP2* expression failed to be directly associated with the severity of ACE, which, conversely, significantly predicted PTSD symptoms selectively in women (β = 0.53, *p* < 0.001). Of note, the total indirect effect of *MECP2* expression on PTSD symptoms was significant in the female (β = −0.14, *p* = 0.033), but not in the male subsample (see Table 3 and Supplementary Table S5 for further details).

## Discussion

The present findings provide evidence of an association between the epigenetic factor MECP2 and symptom severity in traumatized individuals diagnosed with PTSD, a mental illness with severe impact on quality of life and high cost to the health care system [5]. This association appears to occur especially among women, who are typically most affected by PTSD, and is mediated by the quality of early life experiences. These findings suggest that MECP2 may represent a key sex-dependent player in PTSD pathogenesis, and point to *MECP2* expression as a putative marker of vulnerability to stress and trauma–related disorders. Further studies dissecting the underlying mechanisms may unravel targetable pathways for sex-specific preventive interventions.

Previous evidence from the preclinical setting support the existence of a tight link between MECP2 and early life events, and point to MECP2 as a key transducer of perinatal experiences into lasting epigenetic signatures, which ultimately modulate an individual’s ability to cope with future challenges [19, 21, 41, 42, 63–66]. We recently demonstrated the translatability of this framework to the human species, by evidencing an association between *MECP2* levels and subclinical symptoms of anxiety and depression after ACE [43]. The present findings substantiate and transfer this link to a clinical framework by showing that the connection between the severity of PTSD symptoms and *MECP2* levels in traumatized individuals is significantly influenced by exposure to ACE. Indeed, *MECP2* downregulation was related to reporting more ACE and the associated onset of severe PTSD symptoms following exposure to an index trauma in adulthood. Conversely, *MECP2* levels had no connection with the exacerbation of PTSD symptoms linked to ongoing chronic stressors in traumatized participants. An intriguing hypothesis explaining the specific influence of ACE on the link between *MECP2* and PTSD vulnerability concerns the possibility that *MECP2* downregulation may blur the participants’ recall of childhood experiences, without affecting current stress perception, which is in line with the key role exerted by *MECP2* in cognition and memory processes [18]. However, *MECP2* downregulation might also be an immediate consequence of the experience of more ACE. Further studies are certainly needed to ultimately delineate the precise nature of the relationship between MECP2 and early life events.

An intriguing aspect of the present results is that exposure to stressors of varying intensity in different periods of life is accompanied by sex-specific patterns of *MECP2* expression. In fact, women reporting ACE display reduced levels of *MECP2*, while men exposed to traumas in adulthood show *MECP2* overexpression. These results are in line with the evidence describing MECP2 as a critical environmental sensor [19, 67] and suggest that peripheral *MECP2* expression may “quantify” lifetime stress exposure, possibly representing a sex-specific biomarker of vulnerability [68]. Our results in fact point to females as the sex most affected by reduced levels of *MECP2* and the associated negative outcomes of early life challenges. Although the small sample size imposes a cautious interpretation of the data, present results are in line with our previous findings of a sex-dependent association of MECP2 with vulnerability to psychopathology in healthy individuals exposed to ACE [43]. Consistently, females were described to be more vulnerable than males to the detrimental and lasting consequences of ACE [69, 70]. It is thus conceivable that *MECP2* may take part in sex-dependent biological mechanisms that make females more vulnerable than males to stress-related disorders [71]. In this line, other factors that lie within the MECP2 network have been associated with PTSD in a sex-specific manner across rodents and people (e.g. *FKBP5*, *HDAC4*) [72, 73] Furthermore, it is important to underline that developmental sex differences in *MECP2* expression and its contribution to the emergence of sex dimorphisms have been previously acknowledged [37, 38]. The fact that these dimorphisms interest brain regions implicated in PTSD [72] further strengthen our hypothesis of an involvement of MECP2 in the establishment of sex differences in vulnerability to traumas. Gaining further insight into the mechanisms involved in the sex-specific regulation of *MECP2* expression after exposure to stressful experiences will be of great help for the identification of vulnerability or pathogenic pathways to be targeted with the aim of increasing resilience.

The present results should be considered in light of some limitations. Indeed, participants were mainly of European ancestry, thus limiting the possibility to extend our findings to multiple ethnicities. This is important, given that the genetic and epigenetic underpinnings of PTSD have previously been demonstrated to differ among ethnic groups [74]. Moreover, although several studies point to a major involvement of altered DNA methylation processing as a fundamental mechanism providing vulnerability to traumas [7, 12, 40], within the present study we cannot draw conclusions about the functionality of the methylation machinery in patients with PTSD. Further studies are needed to explore the expression of other enzymes involved in DNA methylation and assess MECP2 protein levels, which would be of interest to unveil a functional role for MECP2 and the methylation machinery in the periphery. In this context, it is relevant to clarify that, while analyzing human blood samples allowed us to provide novel evidence of an association between *MECP2* levels and PTSD symptoms, gene expression in blood does not necessarily reflect the molecular processes that may take place within the brain. Although there is evidence that peripheral epigenetic responses might, in some instances, reflect brain-related states [11, 39, 75], the present findings need to be reinforced by animal studies addressing brain *MECP2* levels.

Beyond these considerations, the evidence of an existing link, in the clinical setting, between *MECP2* and the negative outcome of ACE looks promising in the search for vulnerability markers. Indeed ACE represent a risk factor common to multiple mental disorders, including depression and schizophrenia [76, 77], with whom PTSD shares a substantial proportion of genetic variance [10, 78, 79]. In this light, a better understanding of the role of MECP2 in the pathophysiology of mental disorders may benefit from a research focusing on pathological traits, rather than on strict diagnostic categories [80].

Collectively, the present study suggests that *MECP2* downregulation may represent a step in the pathogenic process leading to PTSD onset in patients, especially women, exposed to childhood adversities. Studies focusing on dissecting the mechanisms involved in the regulation of *MECP2* expression could shed new light on the biological pathways underlying the sex and gender bias in trauma vulnerability and could provide a more detailed mechanistic understanding of the pathophysiology of the disorder, hopefully leading to more effective, individualized interventions.

## Supplementary information

Supplementary information

## Data Availability

Ethical restrictions to protect participant confidentiality prevent us from making anonymised study data publicly available. Readers seeking access to the study data and materials should contact the corresponding author based on a formal collaboration agreement. This formal collaboration agreement indicates that data will be shared with other researchers who agree to work with the authors, and for the sole purpose of verifying the claims in the paper. The data and materials will be released to requestors after approval of this formal collaboration agreement by the local Ethics Committee of the Medical Faculty Mannheim.
