# Supplementary material for: Methyl-CpG binding protein 2 expression is associated with symptom severity in patients with PTSD in a sex-dependent manner
Source: Transl Psychiatry. 2023 Jul 7;13:249. doi: 10.1038/s41398-023-02529-9 (PMC10329035; doi:10.1038/s41398-023-02529-9)
Supplement: Supplementary file 1 — Supplementary information [file 41398_2023_2529_MOESM1_ESM.pdf]

Supplement to:

Methyl-CpG binding protein 2 expression is associated with symptom severity in patients with PTSD in a sex-dependent manner

Supplementary methods

Study participants

Participants received a reimbursement for participation (10€/h) plus travel expenses and patients were offered treatment in the clinics of the Central Institute of Mental Health in Mannheim. Data on subsamples of this combined sample were reported in [1–4]. No significant differences were observed for age or education among groups. Predictably, participants diagnosed with post-traumatic stress disorder (PTSD) (37 subjects, of which 17 women) scored higher in the Center for Epidemiological Studies Depression Scale (CES-D) [5] and in the trait version of the State-Trait Anxiety Inventory (STAI-T) [6] measuring depressive and anxiety symptoms, respectively. PTSD patients reported higher levels of chronic stress, measured by the means of the Trier Inventory for Chronic Stress (TICS) [7] and of childhood adversities, as demonstrated by the significantly higher scores obtained in the Childhood Trauma Questionnaire (CTQ) [8]. See Table 1 for further details.

Psychometric measures

Structured Clinical Interviews for DSM-IV. PTSD diagnosis and possible comorbidities were evaluated by the Structured Clinical Interviews for axis I and axis II disorders according to the DSM-IV (SCID-I and SCID-II) [9,10]. They are both semi-structured clinical interviews, administered by trained clinicians, designed to yield diagnoses of mental disorders consistent with DSM- IV [11] diagnostic criteria for major mental and personality disorders.

State-Trait Anxiety Inventory. Trait anxiety was evaluated by the means of the trait scale of the German version of the State-Trait Anxiety Inventory (STAI-T) [6], which is composed of 20 questions rated on a 4-point Likert scale ranging from 1 - nearly never to 4 - nearly always. Scores range from 20 to 80, with higher scores indicating greater trait anxiety.

Center for Epidemiological studies Depression Scale. Depressive symptoms were assessed using the German version of the Center for Epidemiological Studies Depression Scale (CES-D; German Allgemeine Depressions Skala, ADS) [5], a self-report instrument assessing the frequency of 20 depressive symptoms in the last week using a 4-point scale ranging from 0 - rare to 3 - mostly. The score ranges from 0 to 60.

Statistical Analyses

Data distribution was assessed evaluating skewedness and kurtosis; in case of asymmetry a logarithmic transformation was performed to reduce skewedness and kurtosis.

Supplementary tables

Table S1

Data transformation
Logarithmic transformation was performed to reduce the skewedness and kurtosis of the distribution of adverse childhood experiences (ACE). [Abbreviations: SD - standard deviation].

	Non-transformed			Log_{10} transformed		
	**Mean±SD**	**Skewedness**	**Kurtosis**	**Mean±SD**	**Skewedness**	**Curtosis**
**ACE**	36.53±1.61	1.47	2.06	1.54±0.02	0.66	-0.01

Table S2

Direct and Indirect effects in the hypothesized model
[Abbreviations: *MECP2* - methyl-CpG binding protein 2; PTSD – post-traumatic stress disorder; b - unstandardized coefficient; SE - standard error; CI – 95% confidence intervals for b; β - standardized coefficient. Symbols: → - direct effects; →→ - indirect effects; underlined – significant results].

	**b (SE)**	**CI**	**β**	**p-value**
MECP2 → childhood adversities	-0.13 (0.06)	-0.26 – 0	-0.24	0.037
childhood adversities → PTSD symptoms	1.58 (0.69)	0.16 – 3.11	0.27	0.022
MECP2 →→ PTSD symptoms	-0.20 (0.14)	-0.58 – -0.01	-0.06	0.037

Table S3

Direct and Indirect effects in the PTSD symptom scale substitution model
[Abbreviations: *MECP2* - methyl-CpG binding protein 2; PTSD – post-traumatic stress disorder; b - unstandardized coefficient; SE - standard error; CI – 95% confidence intervals for b; β - standardized coefficient. Symbols: → - direct effects; →→ - indirect effects; underlined – significant results].

	**b (SE)**	**CI**	**β**	**p-value**
MECP2 → childhood adversities	-0.16 (0.07)	-0.29 – -0.03	-0.27	0.015
childhood adversities → PTSD symptoms	2.85 (0.70)	1.56 – 4.02	0.44	<0.001
MECP2 →→ PTSD symptoms	-0.45 (0.21)	-0.91 – -0.11	-0.12	0.009

Table S4

Direct and Indirect effects in the chronic stress model
[Abbreviations: *MECP2* - methyl-CpG binding protein 2; PTSD – post-traumatic stress disorder; b - unstandardized coefficient; SE - standard error; CI – 95% confidence intervals for b; β - standardized coefficient. Symbols: → - direct effects; →→ - indirect effects; underlined – significant results].

	**b (SE)**	**CI**	**β**	**p-value**
MECP2 → chronic stress	0.12 (0.30)	-0.47 – 0.71	0.05	0.697
Chronic stress → PTSD symptoms	0.64 (0.14)	0.40 – 0.92	0.51	<0.001
MECP2 →→ PTSD symptoms	0.07 (0.19)	-0.33 – 0.45	0.02	0.685

Table S5

Direct and Indirect effects in men and women subsamples

[Abbreviations: *MECP2* - methyl-CpG binding protein 2; PTSD – post-traumatic stress disorder; b - unstandardized coefficient; SE - standard error; CI – 95% confidence intervals for b; β - standardized coefficient. Symbols: → - direct effects; →→ - indirect effects; underlined – significant results].

	men				women			
	**b (SE)**	**CI**	**β**	**p**	**b (SE)**	**CI**	**β**	**p**
MECP2 → childhood adversities	-0.04 (0.09)	-0.27 – 0.15	-0.06	0.702	-0.22 (0.14)	-0.44 – 0.01	-0.26	0.111
childhood adversities → PTSD symptoms	0.08 (1.09)	-2.34 – 2.47	0.01	0.941	2.89 (0.88)	1.04 – 5.09	0.53	<0.001
MECP2 →→ PTSD symptoms	-0.003 (0.138)	-0.36 – 0.26	-0.001	0.950	-0.63 (0.33)	-1.47 – -0.10	-0.14	0.033

Supplementary figures

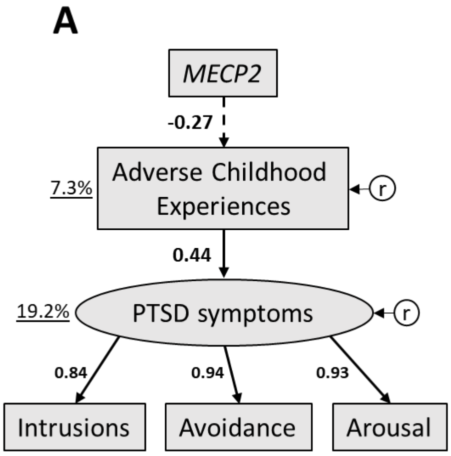

Figure S1 – Reduced MECP2 expression is linked with the increase in PTSD symptom severity associated with ACE exposure but not current stress load in traumatized participants. **A)** Reduced *methyl-CpG binding protein 2* (*MECP2*) expression is directly associated with higher adverse childhood experiences (ACE) scores, which in turn predict increased post-traumatic stress disorder (PTSD) symptom severity. **B)** *MECP2* is not significantly associated with chronic stress load, which predicts increased PTSD symptom severity. PTSD symptom severity is measured with the total score of the Posttraumatic Diagnostic Scale (PDS) (A) or the clinician-administered PTSD scale (CAPS) (B). Symbols: → directed arcs (nonsignificant); → directed arcs, positive path coefficients ($p<0.05$); ---→ directed arcs, negative path coefficients ($p<0.05$). **black numbers** - standardized coefficients; black underlined numbers – proportion of total variation explained by the model (R^2); r – residual variances (errors).

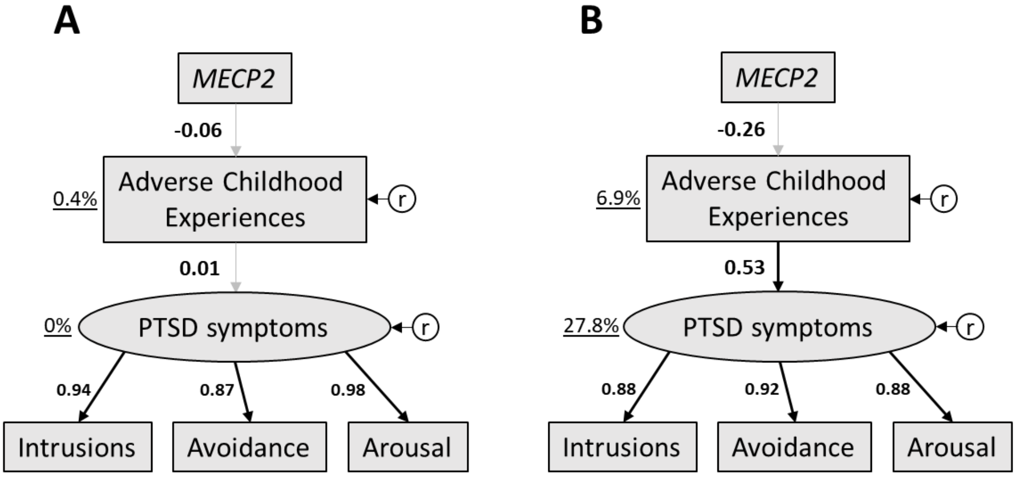

Figure S2 – Reduced MECP2 expression is linked with the increase in PTSD symptom severity associated with ACE exposure selectively in traumatized women. Expression of *methyl-CpG binding protein 2* (*MECP2*) is indirectly associated with post-traumatic stress disorder (PTSD) symptomatology via the mediation of childhood adverse experiences in women (B), but not in men (A). PTSD symptom severity is measured with the total score of the clinician-administered PTSD scale (CAPS). Symbols: → directed arcs (nonsignificant); → directed arcs, positive path coefficients ($p<0.05$); ---→ directed arcs, negative path coefficients ($p<0.05$). **black numbers** - standardized coefficients; black underlined numbers – proportion of total variation explained by the model (R^2); r – residual variances (errors).

Supplementary references

1 Siehl S, Wicking M, Pohlack S, Winkelmann T, Zidda F, Steiger-White F *et al.* Structural white and gray matter differences in a large sample of patients with Posttraumatic Stress Disorder and a healthy and trauma-exposed control group: Diffusion tensor imaging and region-based morphometry. *NeuroImage Clin* 2020; **28**: 102424.

2 Wicking M, Steiger F, Nees F, Diener SJ, Grimm O, Ruttorf M *et al.* Deficient fear extinction memory in posttraumatic stress disorder. *Neurobiol Learn Mem* 2016; **136**: 116–126.

3 Steiger F, Nees F, Wicking M, Lang S, Flor H. Behavioral and central correlates of contextual fear learning and contextual modulation of cued fear in posttraumatic stress disorder. *Int J Psychophysiol* 2015; **98**: 584–93.

4 Siehl S, Wicking M, Pohlack S, Winkelmann T, Zidda F, Steiger-White F *et al.* Altered frontolimbic activity during virtual reality-based contextual fear learning in patients with posttraumatic stress disorder. *medRxiv* 2022. doi:10.1101/2022.06.07.22275758.

5 Hautzinger M, Bailer M. *Allgemeine Depressions-Skala [General Depression-Scale]*. Hogrefe; Göttingen, 1993.

6 Spielberger CD, Gorsuch RL, Lushene RE. *STAI manual for the state-trait anxiety inventory. Self-Evaluation Questionnaire*. Consulting Psychologists Press, 1970.

7 Schultz P, Schlotz W. Trierer Inventar zur Erfassung von chronischem Stress (TICS): Skalenkonstruktion, teststatistische Überprüfung und Validierung der Skala Arbeitsüberlastung [The Trier Inventory for the Assessment of Chronic Stress (TICS)]. *Diagnostica* 1999; **45**: 8–19.

8 Bernstein D, Fink L. *Childhood Trauma Questionnaire: a retrospective self-report (manual)*. 1998.

9 Fydrich T, Renneberg B, Schmitz B, Wittchen H. *Strukturiertes Klinisches Interview für DSM-IV Achse II: Persönlichkeitsstörungen (SKID-II) [Structured clinical interview for DSM-IV, Axis II: Personality disorders]*. Göttingen: Hogrefe, 1997.

10 Wittchen HU, Wunderlich U, Gruschwitz S, Zaudig M. *Strukturiertes klinisches Interview für DSM-IV, Achse I: Psychische Störungen (SKID-I) [Structured clinical interview for DSM-IV, Axis I: Mental disorders]*. Göttingen: Hogrefe, 1997.

11 American Psychiatric Association. *Diagnostic and Statistical Manual of Mental Disorders, Fourth Edition, Text Revision (DSM-IV-TR) (Vol. 1)*. 2000 doi:10.1176/appi.books.9780890423349.
